# *rmc-discord*: reverse Monte Carlo refinement of diffuse scattering and correlated disorder from single crystals

**DOI:** 10.1107/S1600576721010141

**Published:** 2021-11-23

**Authors:** Zachary J. Morgan, Haidong D. Zhou, Bryan C. Chakoumakos, Feng Ye

**Affiliations:** a Neutron Scattering Division, Oak Ridge National Laboratory, Oak Ridge, TN 37831, USA; b Department of Physics and Astronomy, University of Tennessee, Knoxville, TN 37996, USA

**Keywords:** diffuse scattering, correlated disorder, single crystals, *rmc-discord*, Monte Carlo refinement, supercells

## Abstract

A user-friendly program has been developed to analyze diffuse scattering from single crystals with the reverse Monte Carlo method. The approach allows for refinement of correlated disorder from atomistic supercells with magnetic or structural (occupational and/or displacive) disorder. The program is written in Python and optimized for performance and efficiency. Refinements of two user cases obtained with legacy neutron-scattering data demonstrate the effectiveness of the approach and the developed program. It is shown with bixbyite, a naturally occurring magnetic mineral, that the calculated three-dimensional spin-pair correlations are resolved with finer real-space resolution compared with the pair distribution function calculated directly from the reciprocal-space pattern. With the triangular lattice Ba_3_Co_2_O_6_(CO_3_)_0.7_, refinements of occupational and displacive disorder are combined to extract the one-dimensional intra-chain correlations of carbonate molecules that move toward neighboring vacant sites to accommodate strain induced by electrostatic interactions. The program is packaged with a graphical user interface and extensible to serve the needs of single-crystal diffractometer instruments that collect diffuse-scattering data.

## Abstract 2

A user-friendly Python-based program has been developed to analyze diffuse scattering from single crystals with the reverse Monte Carlo method. The approach allows for refinement of correlated disorder from atomistic supercells with magnetic or structural (occupational and/or displacive) disorder.

## 1. Introduction

Crystalline materials with disorder giving rise to unusual properties can be studied with diffuse-scattering measurements (Frey, 1995 ▸). These behaviors involve a wide range of phenomena from magnetically to structurally disordered crystals. Quantifying and understanding the diffuse scattering is crucial to developing property–structure relationships of the disorder in crystalline materials. Unlike conventional crystallography and diffraction in which the ‘average’ structure is obtained, diffuse-scattering studies reveal the local ‘deviations’ away from the average (Welberry & Butler, 1995 ▸). Often, the disorder is locally correlated, involving one or more of the three general disorder types. These include correlations of magnetic moments, site occupancies and/or atomic dis­place­ments.

Correlated disorder is essentially ubiquitous in all functional materials, and the nature of that disorder is closely related to its particular property functionalities (Keen & Goodwin, 2015 ▸). Diffuse scattering can be measured in both powder and single-crystal diffraction experiments; however, characterization of the three-dimensional reciprocal space is achievable by instrumentation advancements that allow complete volumes of single-crystal diffraction data to be collected (Welberry & Goossens, 2014 ▸). A recent technique has emerged to obtain the three-dimensional ‘difference’ pair distribution function (3D-ΔPDF) from single-crystal diffuse-scattering data. This autocorrelation function describing the deviations away from the average structure can be obtained for occupational/displacive disorder (Weber & Simonov, 2012 ▸) and magnetic disorder (Roth *et al.*, 2018 ▸) through the Fourier transform. The refinement program *Yell* (Simonov *et al.*, 2014 ▸) is capable of refining the disordered structure from the full-volume diffuse-scattering data set interpreting with the 3D-ΔPDF.

Although powerful, the 3D-ΔPDF requires special care to separate the Bragg and diffuse scattering from the total scattering signal. For example, Bragg reflections are removed and the missing data are filled back in by interpolation to resemble the surrounding diffuse intensity data. However, this process may introduce artefacts in correlations with larger separation vectors. More advanced methods to deal with Bragg peaks include *K*-space algorithmic reconstruction (*KAREN*), which uses statistical methods to separate the Bragg from the diffuse scattering (Weng *et al.*, 2020 ▸). Another challenge with the 3D-ΔPDF is the requirement of a complete volume of diffraction data. Often, it is impossible to obtain complete reciprocal-space volumes so data symmetrization is utilized (Michels-Clark *et al.*, 2016 ▸). In many instances, the number of available symmetry operators is reduced by the disorder itself. Similarly, sample environments on instruments limit the detector coverage, further impinging the volume mapping. In these cases, alternative approaches to model-free methods are considered to obtain the three-dimensional correlations.

Atomistic Monte Carlo methods are one such technique, where discrete crystals are constructed with atoms located at their average positions within a unit cell (Proffen & Welberry, 1998*b* ▸). A supercell is generated by extending the unit cell in three-dimensional space, and disorder is introduced by decorating atoms with either magnetic moments, vacancies or atomic displacements. Changes are made by rotating moments, replacing atoms with vacancies and displacing atoms until the disordered supercell configuration agrees with experiment through the calculated diffraction intensity. This relatively straightforward approach is a robust method for quantifying the disorder from experimental single-crystal diffraction data, allowing for direct calculation of three-dimensional pair correlations.

Using forward Monte Carlo (FMC) modeling, a Hamiltonian is constructed that describes the system energy and involves coupling constants between the neighboring atoms (Welberry & Butler, 1994 ▸). For example, a general Heisenberg model (Ashcroft & Mermin, 1976 ▸) is used in magnetically disordered systems where each spin vector pair has an (isotropic or anisotropic) exchange coefficient describing the interaction strength between bonds. To simplify the Hamiltonian, often pairs up to a finite number of nearest neighbors are considered. In addition, long-range dipole interactions are greatly simplified assuming Ising spin constraints along easy directions such as in spin ice (Bramwell & Gingras, 2001 ▸). Similar simple energy models are used to capture occupational disorder using binary random variables and displacive disorder using harmonic potentials (Proffen, 2000 ▸). As these simplified models generate many of the diffuse-scattering features (Welberry, 2004 ▸), additional effort is required for more complicated crystal structures where the models become more difficult to adapt and the disorder is not well known or established.

The other atomistic method is reverse Monte Carlo (RMC). RMC is characterized not by an energy function with a model but rather by a goodness-of-fit parameter used to ‘fit’ the supercell disorder to experimentally obtained scattering data (McGreevy & Pusztai, 1988 ▸; McGreevy, 2001 ▸). RMC does not use a Hamiltonian and is a good tool for systems with unknown disorder. This is generally the case for most complicated material systems that are difficult to model. Several RMC programs exist for analysis of diffuse scattering, including *RMCProfile* for polycrystalline and powder diffraction (Tucker *et al.*, 2007 ▸), *Spinvert* for powder diffraction of magnetic materials (Paddison *et al.*, 2013 ▸), and *DISCUS* for powder and single-crystal diffraction (Proffen & Neder, 1997 ▸).

The analysis of single-crystal diffuse scattering from diverse materials systems with complicated behaviors over different conditions is a serious challenge even with RMC (Nield *et al.*, 1995 ▸). The resources required to perform refinements are computationally intensive since the diffuse-scattering pattern has to be recalculated for each proposed move of the RMC refinement. A user-friendly and efficient single-crystal RMC program that integrates into data-reduction workflows is highly desired at X-ray and neutron-scattering user facilities, since it avoids construction of complicated models and works on partial volumes of the reciprocal space. The new program *rmc-discord* is introduced to address this challenge for each of the chemical, structural and magnetic disorder types, optimizing algorithms for performance and providing a user-friendly experience.

## 2. Reverse Monte Carlo approach

The RMC method uses the Metropolis algorithm, similarly to FMC; details are available elsewhere (Proffen & Welberry, 1997 ▸, 1998*a* ▸; Welberry & Proffen, 1998 ▸). For any candidate move that gives an improved fit χ^2^, defined here as 

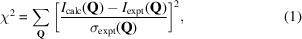

the disturbance is always accepted. Here *I*_calc_(**Q**) and *I*_expt_(**Q**) are the calculated and experimental intensity, respectively. The experimental error σ_expt_(**Q**) is obtained from the data normalization (Michels-Clark *et al.*, 2016 ▸). If instead the fit worsens, the move is either accepted or rejected with some probability according to an acceptance ratio that is a function of the current system temperature and magnitude of the change in fit. As the refinement progresses and overcomes local minima, fewer and fewer candidates that worsen the fit are accepted as it converges to a global minimum.

### 2.1. Single-crystal diffuse-scattering intensity calculations

A major advancement in the calculation of single-crystal diffuse-scattering patterns from atomistic models utilizes the fast Fourier transform (FFT), giving a speedup of at least a factor of one hundred over traditional algorithms in the program *Scatty* (Paddison, 2019 ▸). Although an RMC implementation of this algorithm is tempting to achieve faster refinements, further improvements can be made recognizing that the diffuse-scattering pattern is calculated repeatedly throughout the refinement. By storing intermediate results in memory, it is possible to update the diffuse-scattering pattern with order corresponding to the chosen sizes of the supercell and reciprocal-space volume data set. Therefore, the recalculation of a diffuse-scattering pattern after a given move in the refinement can be achieved in nearly linear complexity. The *Scatty* algorithm using FFT is summarized below for magnetic and structural disorder types (Paddison, 2019 ▸), and then we discuss the modification of storing intermediate results in memory.

The underlying *Scatty* approach is to perform the FFT of the disordered magnetic or structural parameters over the discrete average position vectors of the supercell lattice points for each unique atom site labeled *j* = 0, 1,…. Through vector addition, the average location of an atom in a crystal can be defined in terms of a lattice vector **R** and its basis vector **r**_*j*_. For a supercell with finite size *N*_1_ × *N*_2_ × *N*_3_, these positions can be written in terms of the direct lattice vectors **a**, **b** and **c**, and integer cell coordinates (*R*_1_, *R*_2_, *R*_3_): 

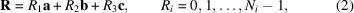

which produces the uniform grid spacing of the discrete Fourier transform (DFT) with (*N*_1_ *N*_2_ *N*_3_)^2^ operations (Briggs & Henson, 1995 ▸). The FFT algorithm efficiently evaluates this using of the order of 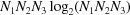 operations. The average position vector **r**_*j*_ of a unique site *j* within the unit cell is written in terms of the direct lattice vectors and its fractional coordinates *u*_*j*_, *v*_*j*_ and *w*_*j*_:

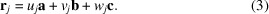

The true position of any atom in the supercell is the sum of its average position and its displacement **u**_*j*_.

Given a real-space field *g*_*j*_(**R**) that captures the disorder among magnetic moments, site occupancies and/or atomic displacements at sites *j* within a cell of the supercell **R**, its DFT is defined by 

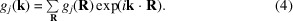

Here, *g*_*j*_(**k**) is the sequence of Fourier coefficients in the spatial frequency domain **k**. This uniformly spaced wavevector is given by 

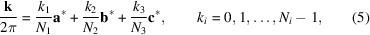

where a*, b* and c* are the primitive reciprocal lattice vectors, and *k*_1_, *k*_2_ and *k*_3_ are grid points. This wavevector **k** is defined within the reciprocal lattice unit cell with resolution determined by the supercell size. To obtain the reciprocal-space wavevector, the reciprocal lattice vector **K** defined in terms of integer Miller indices *h*, *k* and *l*, 

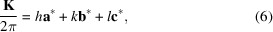

is added to the uniformly spaced wavevector **k**.

The experimental reciprocal-space data set is composed of equally spaced bin sizes and can be written as 

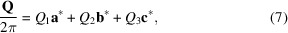

where *Q*_1_, *Q*_2_ and *Q*_3_ are of size *n*_1_ × *n*_2_ × *n*_3_, respectively. Although the supercell size gives the maximum reciprocal-space resolution from **k** defined in equation (5), a finer (or coarser) grid of reciprocal-space points **Q** corresponding to experimentally obtained scattering data can be implemented by using interpolation (or resampling). First, the phase factor in the scattering equations for both magnetic and structural scattering can be written as 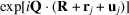 = 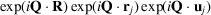 . Recognizing the identity exp(*i* **K** · **R**) = 1, the term exp(*i* **Q** · **R**) ≃ exp[*i*(**K** + **k**) · **R**] = exp(*i* **k** · **R**). The resulting phase factor 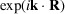 × 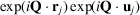 allows for the DFT to be incorporated into the diffuse-scattering calculations that are efficiently evaluated using the FFT algorithm.

The procedure for calculating the diffuse-scattering intensities from a supercell of moments located at their average position (**u**_*j*_ = 0) is summarized below. For magnetic scattering, the DFT of each magnetic moment vector **M**_*j*_(**R**) is computed according to 

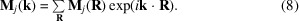

Taking the transformed moments **M**_*j*_(**k**) over each site, the magnetic structure-factor vector **F**(**Q**) is calculated by summing over each unique site *j* within the unit cell: 

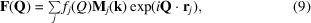

where *f*_*j*_(*Q*) is the magnetic form factor of the ion. The structure factor is then projected into the scattering plane defined by the unit vector 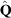 of the reciprocal-space wavevector **Q** using 

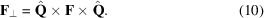

Finally, the magnetic intensity is calculated according to 

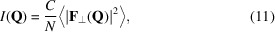

where *C* is a constant (0.07265 b) and *N* is the total number of atoms in the supercell. The angle brackets indicate that multiple supercell refinements can be averaged together (Paddison, 2019 ▸) to reduce the noise of a recalculated pattern. For each individual refinement, however, the refined intensity is calculated using only the refined supercell.

A similar approach is followed for occupational and displacive disorder. Assuming that each site can be occupied by either an atom or a vacancy, a binary site-occupancy parameter δ_*j*_(**R**) can be defined according to 

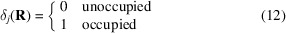

which can be incorporated into the structure-factor calculation. A site that is occupied may also take on some atomic displacement **u**_*j*_(**R**) away from its average position. The structure factor accounting for both occupancy and displacement is given by 

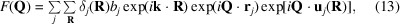

where *b*_*j*_ is the neutron-scattering length of atom *j*. For X-rays, the scattering length is replaced by form factor *f*_*j*_(*Q*). Following the *Scatty* approach, it is necessary to take the Taylor expansion of 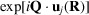 truncated to order *n* about **Q** · **u**_*j*_(**R**) = 0. A second-order expansion is typically sufficient, but higher orders are necessary to capture some diffuse features (Butler & Welberry, 1993 ▸). The trinomial expansion for the powers of the dot product in Cartesian coordinates for the *m*th term of the Taylor series can be written in a condensed notation as 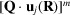 = 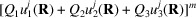 = 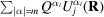 , where α indicates three-dimensional multi-index notation, a three-tuple of non-negative integers α = (α_1_, α_2_, α_3_), and |α| = α_1_ + α_2_ + α_3_. The Taylor expansion products *Q* ^α^ and 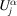 are defined by 

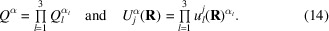

By definition, 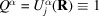 when *m* = 0.

The occupancy parameter and displacement products can be combined to give a new structural parameter. To enforce the overall composition *c*_*j*_ for each atom *j*, the relative occupancy parameter is defined as

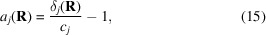

which takes the values *a*_*j*_(**R**) = −1 and *a*_*j*_(**R**) = 1/*c*_*j*_ − 1 when it is unoccupied and occupied, respectively. Multiplying the site-occupancy parameter by the displacement products gives a new structural parameter, 

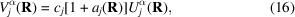

which can be converted to the wavevector domain by DFT, 

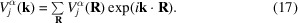

The structure factor in equation (13) can then be approximated by 

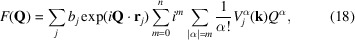

where α! = α_1_!α_2_!α_3_!. Lastly, the diffuse-scattering intensity is determined by 

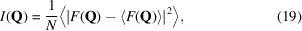

where 〈*F*(**Q**)〉 is the Bragg structure factor (Frey, 1995 ▸; Paddison, 2019 ▸) and the outer angle brackets indicate averaging over multiple refinements. Again, this averaging over supercells is only carried out for the recalculated pattern. In the limit of no displacements, **u**_*j*_(**R**) = 0 and only the *m* = 0 term remains in the Taylor expansion products such that the two right-most sums in equation (18) simplify to δ_*j*_(**R**). Similarly, in the limit of no occupational disorder, δ_*j*_(**R**) = *c*_*j*_[1 + *a*_*j*_(**R**)] = 1, and the parameter simplifies to 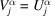 .

### 2.2. Storing of intermediate results in memory

For each RMC move, one atom in the supercell is changed, requiring the complete recalculation of the scattering pattern. A straightforward implementation for recalculating the intensity from the structure factor is to recompute the FFT in 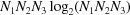 operations for parameters affected by the change at location (*R*_1_, *R*_2_, *R*_3_, *j*). Next, recalculate the structure factor by multiplying the FFT result with phase and form factors summing over each site *j*. Finally, obtain the new intensity from the magnitude of the structure factor. However, the intermediate results for each one of these operations can be computed before the refinement and stored into memory. Significant speedups in recalculating the intensity can be made by taking advantage of these intermediate results, reusing and updating them as needed according to whether a move is accepted or rejected. Using the definition of the DFT, the transformed results of a given field computed initially by FFT can be updated with order corresponding to the supercell size. The structure factors can be updated using a similar procedure with order corresponding to the size of the experimental data set. The two procedures for updating the DFT result and structure factor are outlined below.

Beginning with the DFT update procedure, every proposed move is accompanied by a change in only one atom of the supercell with coordinates (*R*_1_, *R*_2_, *R*_3_, *j*). All other atoms remain unchanged. Considering the field represented by the arbitrary function *g*_*j*_(**R**) = *g*_*j*_(*R*_1_, *R*_2_, *R*_3_) in equation (4), its DFT *g*_*j*_(**k**) = *g*_*j*_(*k*_1_, *k*_2_, *k*_3_) is affected for all *k*_*i*_ = 0, 1,…, *N*_*i*_ − 1. Using the FFT, recalculation of equation (4) reduces the order of operations from (*N*_1_ *N*_2_ *N*_3_)^2^ to 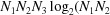 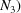 . However, intermediate results from the DFT calculation itself can be stored in memory to update *g*_*j*_(**k**) on the order of *N*_1_ *N*_2_ *N*_3_.

**Figure 1:**
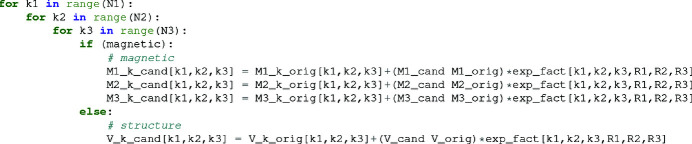
Pseudocode for updating the magnetic and structure Fourier components using the intermediate results. For a given change in the supercell at location (*R*_1_, *R*_2_, *R*_3_, *j*), a nested for loop of size *N*_1_ × *N*_2_ × *N*_3_ is needed to update the Fourier components. By comparison, recalculation of the DFT by FFT requires on the order of 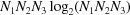 operations.

First, the exponential factors exp(*i* **k** · **R**) are calculated and stored in an array exp_fact[k1,k2,k3,R1,R2,R3] of size (*N*_1_ *N*_2_ *N*_3_)^2^ at the beginning of the refinement. For a chosen atom with coordinates (*R*_1_, *R*_2_, *R*_3_, *j*), the *N*_1_ *N*_2_ *N*_3_ values of *g*_*j*_(*k*_1_, *k*_2_, *k*_3_) are copied into a smaller ‘original’ array for site *j*. A single ‘candidate’ value is generated at (*R*_1_, *R*_2_, *R*_3_, *j*) for *g*_*j*_(*R*_1_, *R*_2_, *R*_3_). Next, a Fourier components candidate array of size *N*_1_ *N*_2_ *N*_3_ corresponding to site *j* is computed by adding to the original array the product of the exponential factors with the difference between the candidate and original value of *g*_*j*_(*R*_1_, *R*_2_, *R*_3_). This is carried out for all *k*_*i*_ = 0, 1,…, *N*_*i*_ − 1. Pseudocode of this procedure is shown in Fig. 1 ▸ for updating the Fourier components of the magnetic moment vector **M**_*j*_(**k**) and the structural parameter 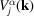 . Once the candidate array is computed, it is then used to update the structure factor.

**Figure 2:**
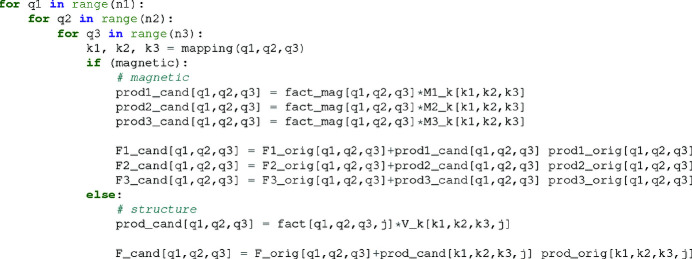
Pseudocode for updating the structure factors using the intermediate results and a nested for loop of size *n*_1_ × *n*_2_ × *n*_3_. Multiplying the updated Fourier components by precomputed fixed factors into a product array, the structure factor is updated by simply adding the difference between the candidate and original arrays. A mapping function is used to account for nearest-neighbor interpolation due to differences in reciprocal-space and Fourier-space resolution.

A similar procedure is utilized to update the structure factors. At the beginning of the refinement, two copies of the structure factor are made. For every proposed update, one is used to calculate the candidate structure factor while the other is used to store its original values. The structure-factor parameters that never change are also stored in an array fact[q1,q2,q3,j]. Here, *q*_1_, *q*_2_ and *q*_3_ are integer indices corresponding to *Q*_1_, *Q*_2_ and *Q*_3_ in equation (7). For magnetic disorder, the factors are 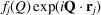 , and for occupational/displacive disorder, the factors are 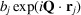 . The products of these factors with all of the corresponding Fourier components are saved in a product array: these products include one for each of the three vector components of the magnetic moment vector **M**_*j*_(**k**) or one for the structural parameter 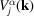 . These arrays have size *n*_1_ *n*_2_ *n*_3_ multiplied by the number of unit-cell atoms. For each update of a Fourier component corresponding to site *j*, the *n*_1_ *n*_2_ *n*_3_ values in the product array for site *j* are copied into an original array before a candidate array is computed by multiplying the Fourier components with the factors array. The candidate structure-factor array is computed for all *Q*_*i*_ by adding to the original array the difference between the candidate and original product arrays. This removes the need to sum over all *j* as in equations (9) and (18). All structure-factor updates can be accomplished in the order of reciprocal-space size. This update procedure is illustrated in Fig. 2 ▸, which displays the pseudocode for the magnetic structure-factor vector **F**(**Q**) and nonmagnetic structure factor *F*(**Q**).

After the candidate structure factors are calculated, the intensities are calculated using equations (11) and (19). The typical RMC refinement continues. If the proposed move is accepted, the candidate Fourier component array is copied into the larger array of components. The candidate products array is also copied into its corresponding larger array. If the move is rejected, no copies are made into the larger arrays and a new move is proposed. This procedure of saving intermediate results allows for fast recalculation of the intensity. It also benefits from multi-threaded parallelism.

### 2.3. Implementation and libraries

The program is written in Python (https://www.python.org/), allowing for efficient development and interfacing with many widely available software libraries including *NumPy* (https://numpy.org/) for array manipulation, *SciPy* (https://www.scipy.org/) for advanced scientific functions like *k*D trees, and *Matplotlib* (https://matplotlib.org/) for plotting. Performance-critical functions and subroutines are written in Cython (https://cython.org/), providing extensions with the performance of compiled C code with support for parallelism through OpenMP (https://www.openmp.org/) with parallel ‘for loops’. To read crystallographic information framework (CIF) files, *PyCifRW* (Hester, 2006 ▸) is utilized, allowing for the creation of crystals. In addition, it allows for the exportation of supercell data for visualization in external viewers like *VESTA* (Momma & Izumi, 2008 ▸). Diffuse-scattering patterns saved in the NeXus data format (Könnecke *et al.*, 2015 ▸) are read using the Python package *nexusformat* (https://github.com/nexpy/nexusformat). A graphical user interface (GUI) is implemented using *PyQt5* (https://riverbankcomputing.com/software/pyqt/). The package is currently available in Windows and Linux distributions through the Python Package Index (PyPI) (https://pypi.org/). A Mac OS/X is planned in a future release.

### 2.4. Capabilities and user requirements

The RMC program is designed to be simple yet robust to help facilitate the analysis of diffuse-scattering measurements. The only major requirements are a CIF file of the average structure of the crystal and a diffuse-scattering measurement data set for refinement. The program currently supports the reading of NeXus files that are built on top of the HDF5 file format. Alternative data readers will also be implemented and supported. After performing the refinement, the user can calculate and visualize correlations. All data sets can be exported for further analysis in external programs.

## 3. Scientific user cases

To illustrate the effectiveness of the RMC method in extracting the correlated disorder from more complicated systems, two user cases are presented below: one for magnetic disorder and a second for structural disorder. The first is from the mineral bixbyite and the other is from a triangular lattice system Ba_3_Co_2_O_6_(CO_3_)_0.7_. The magnetic case is presented first since it has been previously analyzed using the magnetic 3D-ΔPDF method (Roth *et al.*, 2018 ▸, 2019 ▸). The second nonmagnetic structural case has not been previously analyzed; the RMC program is one tool capable of extracting the correlated disorder. Both data sets were collected from the elastic diffuse-scattering spectrometer CORELLI (Ye *et al.*, 2018 ▸).

### 3.1. Magnetic diffuse scattering of frustrated magnet bixbyite

**Figure 3:**
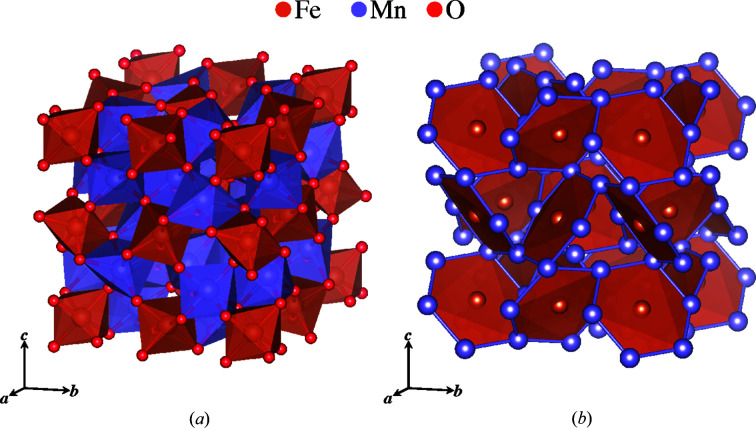
Bixbyite. (*a*) A polyhedral model of (Fe, Mn)_2_O_3_ and (*b*) a network of frustrated hexagons with nearest-neighbor Mn-rich sites surrounding central Fe-rich sites. Below its transition temperature *T* = 32.5 K, bixbyite is highly geometrically frustrated.

The first user case is from the diffuse scattering of single-crystal bixbyite, (Fe, Mn)_2_O_3_, a natural mineral with magnetic iron and manganese ions (Roth *et al.*, 2019 ▸). It is a cubic crystal with space group 206 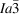 and lattice parameter *a* = 9.409 Å (Pauling & Shappell, 1930 ▸). Shown in Fig. 3 ▸ is the polyhedral model of bixbyite and its network of (Mn, Fe)_2_O_3_ polyhedra. The magnetic Mn^3+^ and Fe^3+^ ions have nearly identical magnetic form factors (Lisher & Forsyth, 1971 ▸) and a similar magnitude of magnetic moments. The two metal sites in the structure are occupied by both manganese and iron: one is Fe rich and the other is Mn rich. The Fe-rich site resides at the origin (0, 0, 0), while the Mn-rich site is located at (0, 0.25, 0.285): that is, nearly (0, 0.25, 0.25). The structure can be thought of as a deviation from a higher-symmetry one with the position of the Mn-rich sites slightly altered. This reduced cubic structure has space group 221 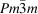 with a lattice parameter that is half of the original. Considering identical ions, the structure corresponds to a face-centered cubic crystal, which itself is a common frustrating magnetic lattice (Ramirez, 1994 ▸).

**Figure 4:**
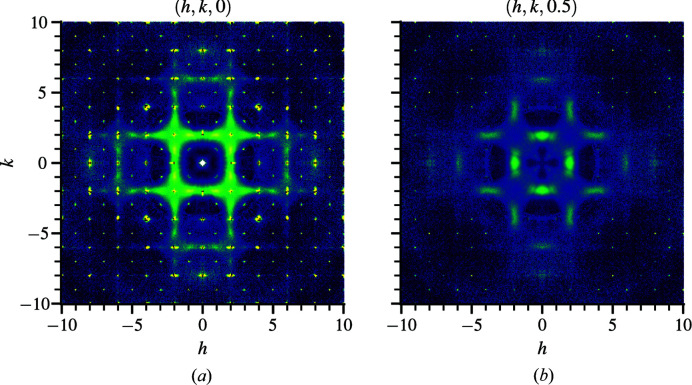
Single-crystal neutron scattering of bixbyite at (*hkl*) planes (*a*) *l* = 0 and (*b*) *l* = 0.5, obtained by subtracting high-temperature *T* = 300 K from low-temperature *T* = 7 K data. The complete reciprocal-space volume covers −10 to 10 in each *h*, *k* and *l* dimension using 501 × 501 × 501 bins. The diffuse-scattering features repeat every four reciprocal lattice units in *h*, *k* and *l*.

For this 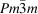 structure, the nearest-neighbor pairs of Fe–Mn and Mn–Mn have identical distances. forming a cuboctahedral network of manganese pairs surrounding a central iron atom. The deviation of Mn-rich sites from the high-symmetry position creates a nearest-neighbor network consisting of a hexagonal arrangement of nearly coplanar manganese pairs surrounding an iron atom, as shown in Fig. 3 ▸(*b*). Bulk property measurements of field-cooled and zero-field-cooled magnetization suggest spin glass behavior with inverse susceptibility indicating strong antiferromagnetic interactions (Roth *et al.*, 2018 ▸). The single-crystal diffuse scattering of bixbyite (Roth *et al.*, 2018 ▸) is displayed in Fig. 4 ▸, which shows Bragg and non-Bragg layers in (*a*) and (*b*), respectively.

A strategy for fast refinement is to focus on a region of the diffuse scattering with minimal overlapping symmetry that covers a primitive segment of the repeating pattern. For bixbyite, the *h*, *k* and *l* range 0–4 satisfies this requirement. In general, a two-dimensional slice is not appropriate for refinements with scattering that show three-dimensional symmetry. Some liberty can be taken to restrict the range of a particular direction while maintaining a good refinement. It is found that the range of one dimension (along *l*) can be reduced by half in bixbyite. However, high and low intensities are both needed for refinement. For any volume not refined against, the program has no way of calculating goodness of fit in those missing regions so the intensity values are unconstrained there. Hence, the primitive features are the ‘minimum’ requirement to obtain a reasonable refinement. A wide coverage with overlapping symmetry is preferred to obtain the best refinement.

**Figure 5:**
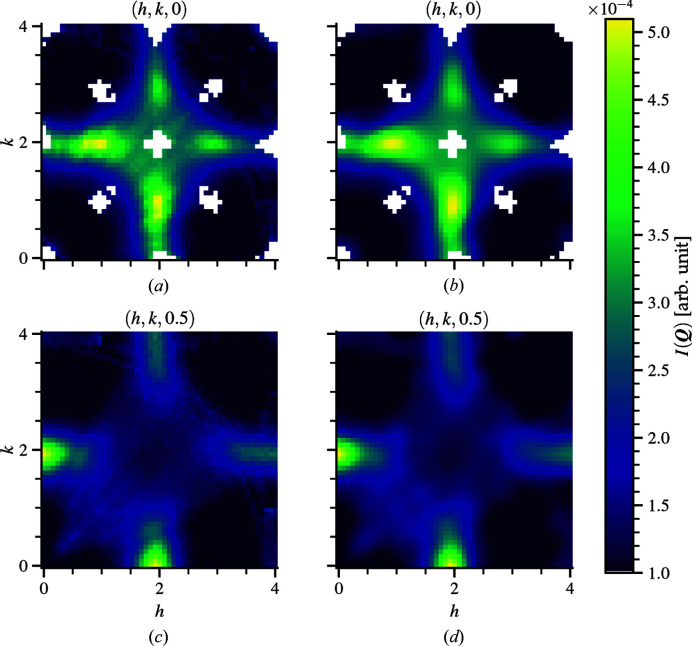
Diffuse-scattering intensity from bixbyite showing selected (*hkl*) planes of the [(*a*), (*c*)] cropped and rebinned experimental data compared with [(*b*), (*d*)] RMC refinement values. The region of interest has *h* and *k* ranging from 0 to 4 and an *l* range from 0 to 2 with a grid size of 0.08. A 4 × 4 × 4 supercell is used. The match between experiment and refinement is excellent. The comparison for plane *l* = 0 with strongest diffuse-scattering features is shown in (*a*) for the experiment data and (*b*) for the refinement values, while (*c*) and (*d*) show the corresponding data for plane *l* = 0.5. The RMC refinement captures all of the subtle features of the experimentally obtained pattern with the nuclear Bragg peaks punched out.

Using the ranges described above, Fig. 5 ▸ shows a comparison between the experimental and refinement data. The Bragg peaks are removed and, unlike for the 3D-ΔPDF, the missing data do not have to be filled back in. Figs. 5 ▸(*a*) and 5 ▸(*b*) shows the comparison between the experiment and refined data, respectively, for a slice taken from the plane *l* = 0. Figs. 5 ▸(*c*) and 5 ▸(*d*) show the corresponding data at slice *l* = 0.5. Both sets of slices are in good agreement. Spin–spin correlations are calculated using the refined supercell structure by taking the dot product between spin vectors of every possible ion pair and averaging the results over common distances and ion pairings within a cutoff distance. With RMC refinement, the subtle differences in pair lengths can be easily resolved in the correlation calculations. As the magnetic 3D-ΔPDF has no underlying information on the average positions of ions within the crystal, it is generally more difficult to distinguish pairs that are closely spaced, especially like they are in bixbyite when considering first- and second-nearest-neighbor manganese pairs.

**Figure 6:**
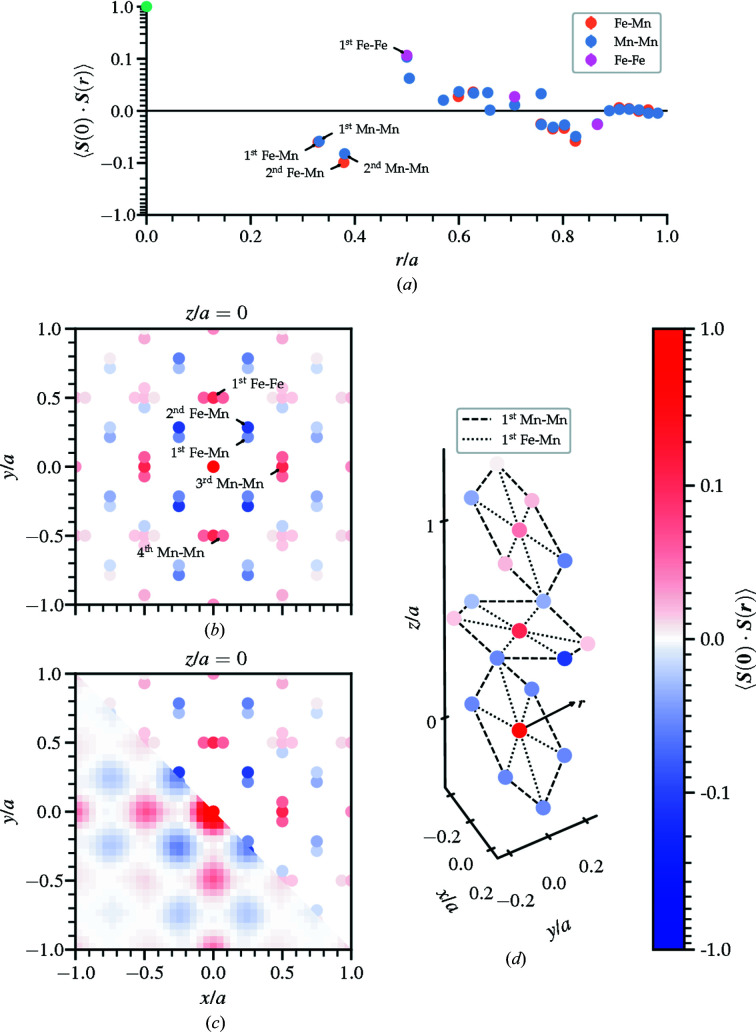
Magnetic spin-pair correlations for bixbyite. (*a*) Spherically averaged radial spin correlations show antiferromagnetic first- and second-nearest-neighbor Fe–Mn (orange fill) and Mn–Mn (blue fill) pairs with the color indicating the bond type. (*b*) The corresponding 3D spin correlations in the *xy* plane with *z* = 0 reveal that the second-nearest-neighbor Fe–Mn antiferromagnetic (blue fill) correlations are stronger than the first. (*c*) The 3D correlations resolve the subtle differences in bond length between neighboring pairs (upper right) better than the magnetic 3D-ΔPDF (lower left). (*d*) A portion of the nearest-neighbor hexagonal network along a principal axis with manganese surrounding iron. Here, iron is placed at the origin (red fill).

The calculated spin–spin correlations for the refinement are plotted in a variety of ways. Fig. 6 ▸(*a*) shows the spherically averaged correlations where the closest nearest-neighbor pairs have strong antiferromagnetic preference. The calculated 3D correlations in the plane *z* = 0 are displayed in (*b*) and compared with the magnetic 3D-ΔPDF in (*c*) using the preprocessed intensity data corresponding to Fig. 4 ▸ by removing Bragg peaks and interpolating the missing data. One advantage of RMC refinement is that the 3D correlations are easily mapped to the proposed frustrated structure. Fig. 6 ▸(*d*) displays the 3D spin-pair correlations corresponding to the nearest-neighbor hexagonal network shown in Fig. 3 ▸(*b*). The calculated spin correlations of Fig. 6 ▸(*a*) are labeled by their corresponding ion pair. It is clear that the six manganese atoms surrounding the central iron atom have antiferro­magnetic preference.

The calculated correlations offer additional detail of pairs with nearly equal separation vectors. This is clear comparing the RMC correlations with the 3D-ΔPDF in Fig. 6 ▸(*c*). For example, the first and second Fe–Mn pairs have slightly different separation vectors deviating from the aforementioned 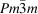 structure. From the refinement, the antiferromagnetic interactions between second-nearest-neighbor pairs are stronger than the first Fe–Mn pairs, the latter of which result in the frustrated hexagonal network. This is also observed in the radial spin correlations plotted in Fig. 6 ▸(*a*), which shows the difference in strength of the antiferromagnetic first and second Fe–Mn pairs. The nearest-neighbor Mn–Mn pairs show similar preference, where the second are stronger than the first. The frustration extends beyond the hexagonal network.

Ion pairs that share the same separation can be individually calculated from the RMC refinement. The first Fe–Fe pairs and third Mn–Mn pairs both share common separation vectors, each apart by half the length of the unit-cell edge. Both of these correlations are strongly ferromagnetic, as indicated in Fig. 6 ▸(*a*). In the context of the hexagonal network, the magnetic moments that reside at the center tend to align with the other central ions of nearby hexagons. Similarly, the magnetic moments along the vertices of the hexagons have alignment preference with the next closest corresponding ion in the neighboring hexagon. The ability to distinguish different ion types allows complicated frustrated structures to be analyzed through the construction of 3D networks, like the one shown in Fig. 6 ▸(*d*), to help guide the building models of the magnetic interactions.

### 3.2. Structural diffuse scattering of disordered Ba_3_Co_2_O_6_(CO_3_)_0.7_

**Figure 7:**
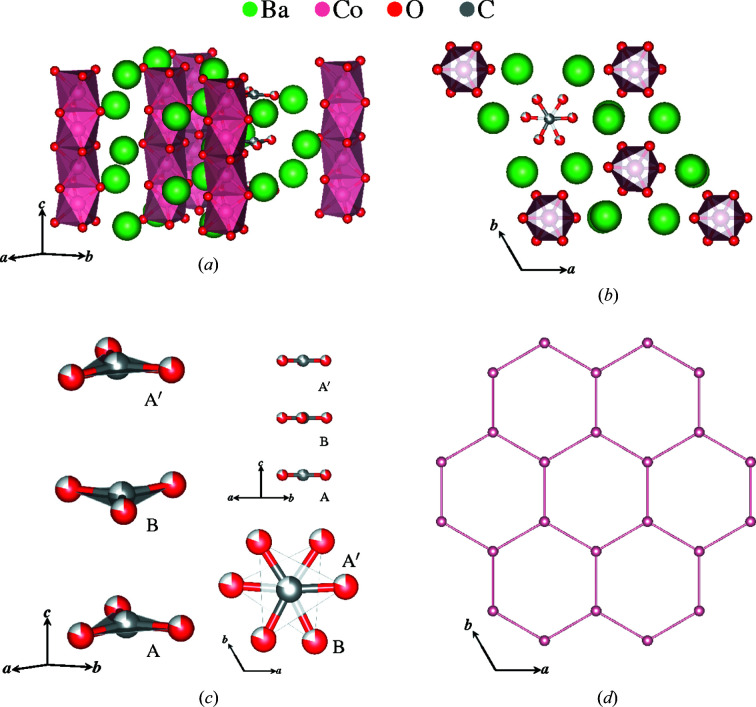
A polyhedral model of Ba_3_Co_2_O_6_(CO_3_)_0.7_ showing chains of CoO_6_ and carbonate CO_3_ in (*a*) the standard orientation and (*b*) along the *c* axis. (*c*) Isolated views of chains of polyatomic CO_3_^2−^ ions with occupancy 0.7. Single-crystal measurements of inverse susceptibility reveal inter-layer ferromagnetic and intra-layer antiferromagnetic interactions that suggest a spin liquid candidate of Ising spins on the (*d*) cobalt honeycomb lattice.

The next example demonstrates RMC refinement of structural disorder from the triangular lattice system Ba_3_Co_2_O_6_(CO_3_)_0.7_. It is a hexagonal crystal with space group 174 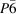 and lattice parameters *a* = 9.683 Å and *c* = 9.518 Å (Boulahya *et al.*, 2000 ▸), composed of CoO_6_ octahedra and carbonate CO_3_ molecular chains along its *c* axis (Iwasaki *et al.*, 2009 ▸), as shown in Figs. 7 ▸(*a*) and 7 ▸(*b*). These chains are visualized in Fig. 7 ▸(*c*), where the average occupancy of the polyatomic ion CO_3_^2−^ molecule is 0.7. The magnetic cobalt ions form honeycomb layers, which is one lattice that can be geometrically frustrated. A single layer is shown in Fig. 7 ▸(*d*).

**Figure 8:**
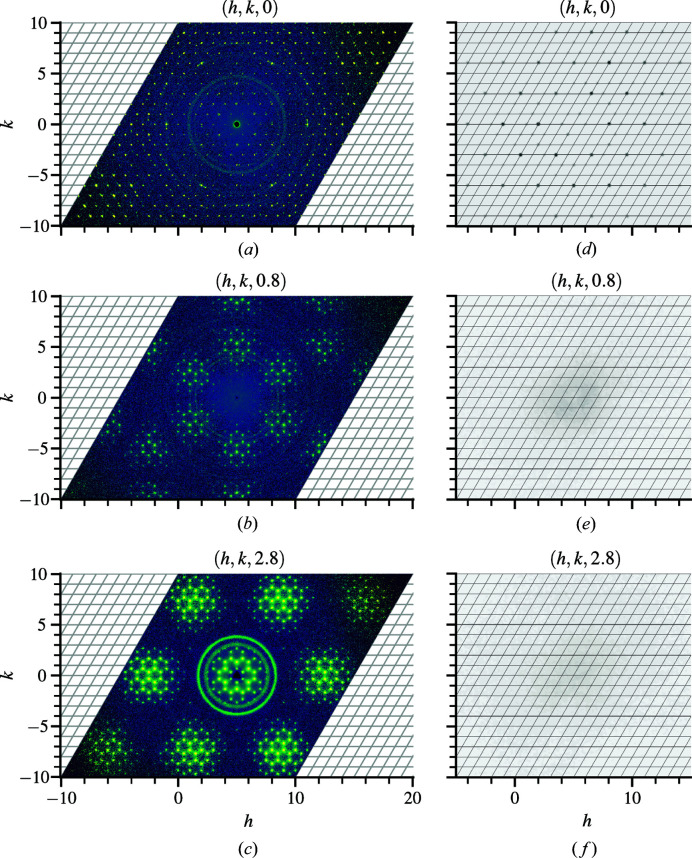
Single-crystal neutron scattering of Ba_3_Co_2_O_6_(CO_3_)_0.7_ at (*hkl*) planes (*a*) *l* = 0.0, (*b*) *l* = 0.8 and (*c*) *l* = 2.8 obtained at *T* = 50 K. The complete reciprocal-space volume covers −10 to 10 in each *h*, *k* and *l* dimension using 501 × 501 × 501 bins. The corresponding single-crystal X-ray scattering data at (*hkl*) planes (*d*) *l* = 0.0, (*e*) *l* = 0.8 and (*f*) *l* = 2.8 obtained at *T* = 150 K from a Rigaku laboratory source do not exhibit any diffuse-scattering features, suggesting that the correlated disorder originates from the light elements which are more sensitive to neutrons than X-rays. For the neutron data, the diffuse scattering remains at temperatures above *T* = 150 K.

The absence of long-range magnetic ordering at low temperatures suggests a spin liquid candidate (Igarashi *et al.*, 2012 ▸); however, the diffuse-neutron-scattering pattern does not show any signs of magnetic correlation even at low temperature. The characteristic drop off in intensity at larger scattering vectors indicative of the magnetic form factor is not present. Instead, diffuse features are observed within different non-integer *l* planes, as shown in Fig. 8 ▸, that persist all the way to room temperature. The Bragg layer at (*a*) *l* = 0 shows no diffuse features, but such features are clearly observed in non-integer layers (*b*) *l* = 0.8 and (*c*) *l* = 2.8. The correlated disorder is therefore along the *c* axis of the crystal and its origin is structural rather than magnetic.

**Figure 9:**
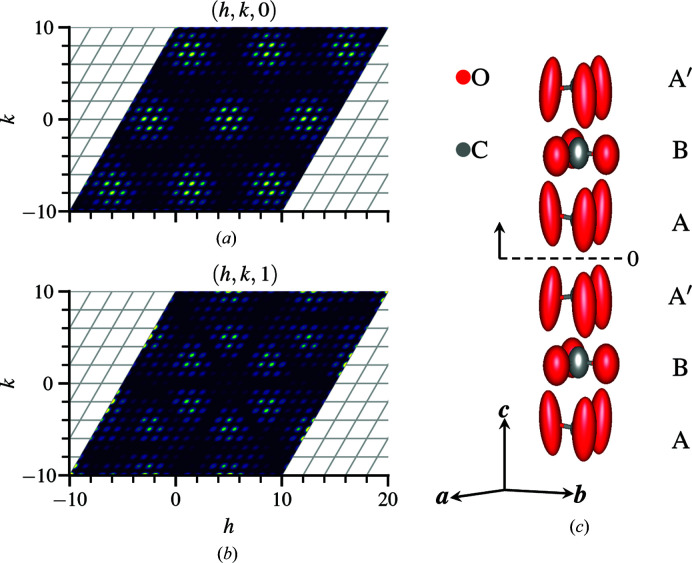
The calculated CO_3_-only Bragg scattering intensity at (*hkl*) planes (*a*) *l* = 0 and (*b*) *l* = 1 resembles the diffuse neutron scattering at (*hkl*) planes *l* = 2.8 and *l* = 0.8, respectively, suggesting that the diffuse pattern originates from CO_3_ disorder along the *c* axis. Additional neutron-scattering data of (*c*) atomic displacement ellipsoids (99% probability) for the carbon and oxygen atoms on the CO_3_ molecule elongated along the *c* axis further hint at correlated displacements of the CO_3_ molecules. The orientation variants are labeled as A, B and A′.

Single-crystal X-ray diffraction measurements with a Rigaku laboratory source reveal no diffuse scattering at these planes, as indicated in Figs. 8 ▸(*d*)–8 ▸(*f*). Because X-ray scattering length is proportional to atomic number (Seltzer, 1995 ▸), the absence of diffuse scattering in the X-ray data would suggest that the heavier elements are not the origin as the neutron-scattering lengths of oxygen and carbon are similar to those of barium and cobalt (Sears, 1992 ▸). Synchrotron measurements could help confirm this. Since the average occupancy of CO_3_ is 0.7, occupational disorder of the molecule itself is one possible origin. Calculating the structure factor of CO_3_ only, it is observed that the Bragg scattering intensity at *l* = 0 and *l* = 1 in Figs. 9 ▸(*a*) and 9 ▸(*b*) resembles that of the diffuse scattering at the *l* = 2.8 and *l* = 0.8 planes in Figs. 8 ▸(*b*) and 8 ▸(*c*), respectively. This indicates that the CO_3_ groups are responsible for the disorder. Neutron-diffraction data collected using the TOPAZ instrument (Schultz *et al.*, 2014 ▸) result in atomic displacement factors with prolate oxygen spheroids generally oriented along the chain, as indicated in Fig. 9 ▸(*c*), suggesting the presence of displacive disorder as well. For these reasons, a detailed analysis of CO_3_ disorder is key to understanding the origin of the diffuse scattering in Ba_3_Co_2_O_6_(CO_3_)_0.7_.

In each unit cell, the three CO_3_ molecules take on one of two orientation variants that are ∼180° from one another. The pattern repeats as A–B–A′–A–B–A′–A–B–A′⋯, where A and A′ have the same orientation distinguished from B with opposite orientation. After RMC refinement, it is straightforward to separate calculated correlations, placing different variants at the origin to investigate the correlated disorder. For molecular-disorder refinement, CO_3_ is kept as a rigid stoichiometric molecule and allowed to be either vacant on a site or present with finite displacement and rotation from its average position and orientation. To further speed up the refinement, the structure factor of the molecule is precalculated and stored as a prefactor. Considering only the displace­ment of the center of mass of the molecule (no rotation), a speedup corresponding to the number of atoms per molecule can be achieved. Such an approach is a general method for handling rigid assemblies of polyhedra.

**Figure 10:**
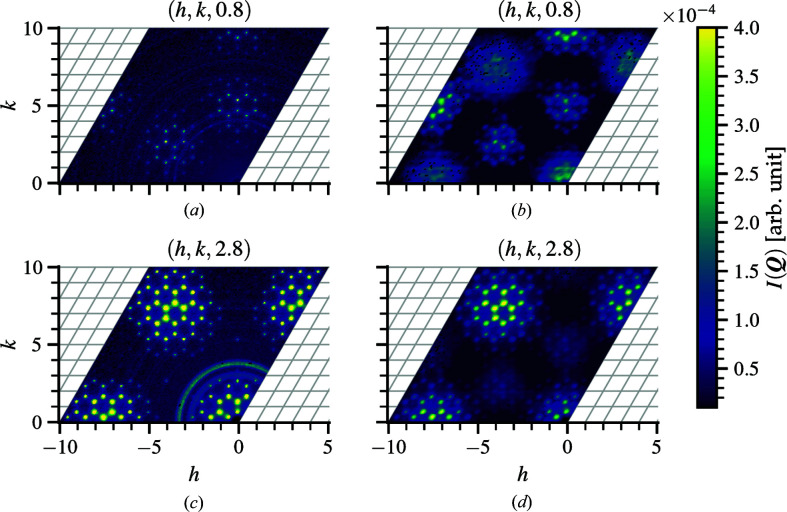
Diffuse-scattering intensity from Ba_3_Co_2_O_6_(CO_3_)_0.7_, showing selected (*hkl*) planes of the [(*a*), (*c*)] cropped and rebinned experimental data compared with [(*b*), (*d*)] RMC refinement values. The region of interest has an *h* range from −10 to 0, a *k* range from 0 to 10, an *l* range from 0 to 4 and a grid size of 0.08. A 12 × 12 × 12 supercell is used. The match between experiment and refinement is good. The comparison for plane *l* = 0.8 is shown in (*a*) for the experiment data and (*b*) for the refinement values, while (*c*) and (*d*) show the corresponding data for plane *l* = 2.8 with the strongest diffuse-scattering features. There are some additional cloudy features in the refined pattern due to the simplified model of rigid molecular displacements. The extra correlations generated by this constraint introduce these artefacts. The RMC refinement is also insensitive to the powder rings associated with the cryomagnet observed in the experimentally obtained pattern as they do not overlap with the main diffuse-scattering features.

Refinement of rigid CO_3_ displacement and occupancy is performed to analyze the structural disorder. Starting with a random distribution of molecules with 70% occupied and no initial displacement, occupational disorder is refined for the first half of the refinement. The remaining molecules are allowed to displace away from their average positions in the second half. Although it is possible to refine both disorder types simultaneously, faster convergence is achieved when the occupational refinement is given more weight earlier in the refinement, since removing a molecule affects the structure factor much more than displacing it. For the displacive refinements, the molecules are allowed to displace anywhere uniformly within a sphere of radius 1.5 Å, which is about half the distance between molecules along the chain. The experimental data set is compared with the resulting refinement pattern obtained with RMC in Fig. 10 ▸: (*a*) and (*b*) compare the plane *l* = 0.8, while (*c*) and (*d*) compare the plane *l* = 2.8. This refinement provides a good fit of the experimental data. Incorporating rotation and distortion of the molecule would help improve the refined pattern. The refined features are also broader due to the finite size of the supercell. Extending its size will improve the sharpness of the refined intensity at the cost of speed. Correlations can then be obtained considering different variants as the origin.

**Figure 11:**
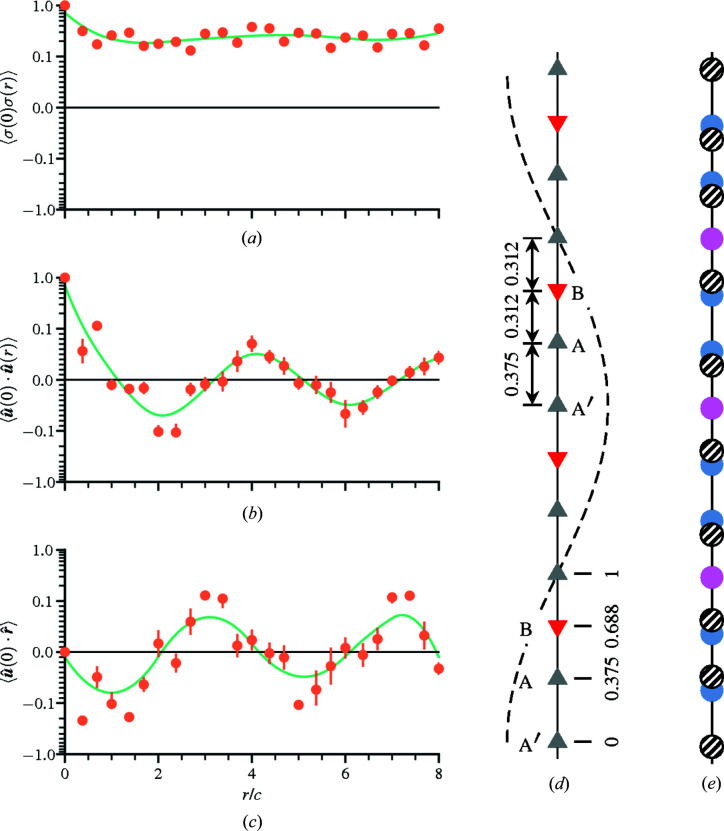
Correlations along the CO_3_ chains of Ba_3_Co_2_O_6_(CO_3_)_0.7_ with the A′ molecule variant located at the origin: (*a*) occupancy, (*b*) displacement and (*c*) vacant–displacement pairs. The chain pair correlations follow the A′–A–B order illustrated in (*d*), the relative unit-cell fractional coordinates and the distance between variants. Representing the molecules as atoms, (*e*) an illustration of chain disorder is shown with displaced molecules (hatch fill) moving away from their average position (blue fill) toward nearby vacant sites (pink fill) along the chain.

Having obtained the occupation and position of the CO_3_, various occupancy, displacement and vacant–displacement pair correlations can be obtained along the molecule chains. For occupational disorder, occupied and vacant molecules are mapped to a binary parameter that is either positive or negative unity, respectively (Welberry & Goossens, 2008 ▸). The calculated occupancy correlations along the chain in Fig. 11 ▸(*a*) are essentially featureless, ruling out any clustering of vacant sites where some characteristic distance by which the correlations become negative would be observed. Considering the average occupancy is 70%, two to three molecules on average are between every pair of vacant sites. The displacement correlations are analogous to magnetic spin-pair correlations or static displacement variables (Welberry & Goossens, 2014 ▸); inspecting them in Fig. 11 ▸(*b*), their modulation corresponds to a periodicity of four unit cells along the chain in the *c*-axis direction. This indicates that, within the first unit cell, neighboring occupied molecules tend to move away from one another.

The interactions between occupational and displacive disorder are quantified by identifying all vacant–occupied pairs. The dot product of the unit separation vector between pairs with the direction vector of the displaced molecule gives a measure of the vacant–displacement correlation. This metric is analogous to an atomic size effect parameter (Welberry, 1986 ▸; Butler *et al.*, 1992 ▸) that quantifies the preference of an atom to move toward or away from a vacancy. In Fig. 11 ▸(*c*), the vacant–displacement correlations have the same periodicity as the displacement correlations but are out of phase by one unit cell. Illustrations of the intra-chain disorder are shown in Figs. 11 ▸(*d*) and 11 ▸(*e*). The spacings between the average positions of the molecules along the chain are nearly equal. As no clustering occurs along the chain, two to three molecules will be present between every pair of vacant sites, and those molecules displace away from their neighbors toward the closest missing site. This reduces the overall spacing between molecules producing the displacement and vacant–displacement modulations. These correlated displacements accommodate the electrostatic interactions between the polyatomic CO_3_ ions and generate the diffuse-scattering pattern observed by neutrons. This behavior of Coulomb field relaxation is similar to another hexagonal system with channeled mol­ecules. For the case of urea inclusion compounds that form a hexagonal network, rigid alkane groups within its channels exhibit orientational disorder (Welberry & Mayo, 1996 ▸) with a repeat distance different from the spacing of the urea itself, which generates diffuse-scattering features in non-integer *l* layers similar to Ba_3_Co_2_O_6_(CO_3_)_0.7_.

## 4. Graphical user interface

An important aspect of the program is its GUI. Although scripts written to perform an RMC refinement are included, they are intended for development of new modules and testing purposes. From the perspective of the user, a simple GUI guides much of the workflow and reduces the learning barrier to obtaining meaningful information from a diffuse-scattering pattern. Four main steps achieve this: (1) building a supercell from the average structure, (2) processing the intensity data to make it suitable for refinement, (3) executing the refinement, and (4) calculating and interpreting the real-space correlations. These major steps are incorporated as tabs of the GUI window.

### 4.1. ‘Crystal’ tab

The ‘Crystal’ tab allows the user to read a CIF file corresponding to the average structure of the crystal being analyzed. In many cases, a user will have a CIF file from preliminary experiments on the sample. If not, several structural databases are available with published CIF files, including the Bilbao Crystallographic Server (Aroyo, Perez-Mato *et al.*, 2006 ▸, 2011 ▸; Aroyo, Kirov *et al.*, 2006 ▸), Cambridge Structural Database (Groom *et al.*, 2016 ▸), Inorganic Crystal Structure Database (Belsky *et al.*, 2002 ▸), Crystallography Open Database (Gražulis *et al.*, 2009 ▸) and others. This generates the unit-cell data including atomic sites, atom positions, atom types and site occupancies without requiring knowledge about the symmetry operators or lattice parameters used to define it. The user can specify the refinement type as either magnetic or nonmagnetic (structural). The size of the supercell can be specified at any time. In addition, the ‘Crystal’ tab allows the unit-cell data to be freely adjusted, including switching atoms on or off, changing occupancies, and manipulating atom types. The program contains many magnetic form factors of ions and neutron-scattering lengths for different isotopes that can be simply selected. Future support for X-ray form factors for structural disorder is expected.

**Figure 12:**
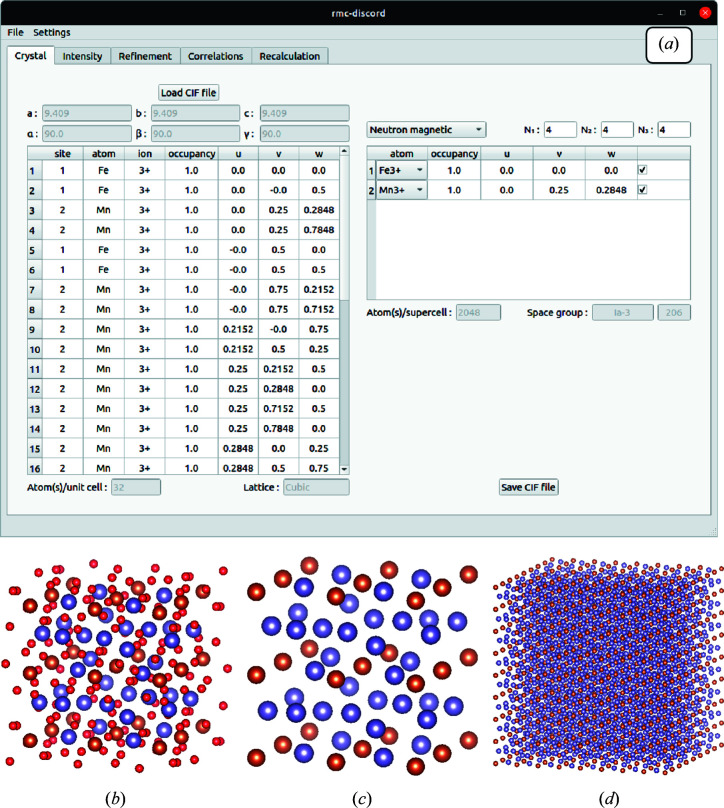
(*a*) The GUI ‘Crystal’ tab for building the supercell for refinement. Illustrations of (*b*) the original unit cell for bixbyite, (*c*) the unit cell with the oxygen atoms removed and (*d*) the final supercell visualized with *VESTA*.

An example of the GUI for bixbyite is given in Fig. 12 ▸. In 12 ▸(*a*), the ‘Crystal’ tab with finalized data used for refinement is shown. The overall unit-cell data are given on the left; a portion of the 32 iron and manganese magnetic ions are shown. The right shows the three atom sites read from the original CIF file. In this case the oxygen atom is unselected as it is not magnetic. Figs. 12 ▸(*b*)–12 ▸(*d*) illustrate this crystal-building process for bixbyite. Loading in the unit-cell data, the oxygen atoms are removed and the supercell is generated by repeating the modified unit cell according to its chosen size. The final supercell may be exported as another CIF file for visualization in an external viewer like *VESTA*.

### 4.2. ‘Intensity’ tab

Once the supercell is generated, the experimental intensity is loaded from a supported data format. Current support is for the NeXus files output by many synchrotron and neutron sources. Future releases will have support for other data formats including *NumPy* arrays. Support for commercial single-crystal X-ray diffractometers is also possible. Once the data set is loaded, the user has options to clean up, crop and rebin the data for refinement. First, the data are rebinned to a coarser bin size using available predetermined sizes from a dropdown menu. The binning sizes available are factors of the original data-set size. The rebinning can be selected for each of the *h*, *k* and *l* dimensions. Next, the data set is cropped to a smaller region of interest by selecting the range for *h*, *k* and *l*.

Bragg peaks may be removed using a punch method. The user selects the cell centering with the default set from the space group within the CIF file. A box or ellipsoid punch size in *h*, *k* and *l* space is specified. Finally, an outlier parameter is defined giving the range of data to be excluded based on the interquartile range of the data within the punch. A default value of 1.5 is the standard definition of an outlier: the parameter (1.5) times the interquartile range excludes the data above and below the third and first quartile, respectively. This is a simple approach to removing Bragg peaks. The punch parameters are adjustable and multiple passes may be performed to give a clean data set for refinement. The punch may be reset at any time. Future implementations of the program may include more advanced algorithms, such as *KAREN* (Weng *et al.*, 2020 ▸) which identifies and removes Bragg peaks from the total scattering without the need for the unit-cell data. In addition, it can remove other anomalies due to sample environment.

**Figure 13:**
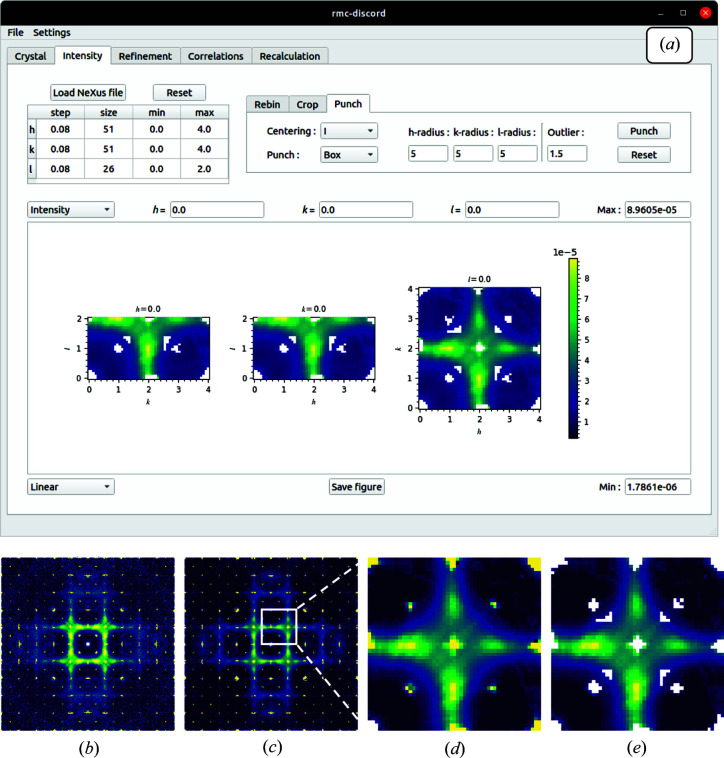
(*a*) The GUI ‘Intensity’ tab for generating raw volume data showing (*b*) the original NeXus file for bixbyite. The figure also shows the data set (*c*) rebinned with a larger bin size, (*d*) cropped to a smaller region and (*e*) with Bragg peaks removed.

The steps for generating clean volume data for refinement are shown in Fig. 13 ▸ for bixbyite: the finalized data set is produced by rebinning, cropping and Bragg-peak removal. The original data set of size 501 × 501 × 501 with *h*, *k* and *l* range −10 to 10 is reduced to a smaller bin size of 0.8 in each dimension, and cropped to a range of 0–4 in *h* and *k* and 0–2 in *l*. The Bragg peaks are then removed in one pass using the default parameters and a box size of five voxels. The resulting data set may be further investigated at different slices and the displayed figure may be saved. At any time during this process, the original data set can be recovered using the reset button.

### 4.3. ‘Refinement’ tab

Having built the supercell and preprocessed the intensity data set, a refinement is now set up. The interface for refinement is flexible with options for both magnetic and structural refinements. The Gaussian filter size may be specified for each of the *h*, *k* and *l* dimensions to reduce the noise in the refined intensity and/or mimic the effects of the instrumental resolution broadening. The number of cycles used for the refinement can be specified. One cycle is defined as the number of proposed moves which corresponds to the number of atoms in the supercell. It is typically best to start with a short refinement (1–10 cycles) to optimize the annealing temperature prefactor and decay constant. In the first 10–20% of the refinement, the temperature should be high enough that all of the proposed moves are accepted. This allows the system to overcome any local minima as it progresses toward a global minimum. During this part of the refinement, the temperature should cool gradually such that some of the bad moves are rejected. By the final 50% of the long refinement (100–200 cycles), nearly all of the bad moves should be rejected as the system is moving toward the global minimum.

**Figure 14:**
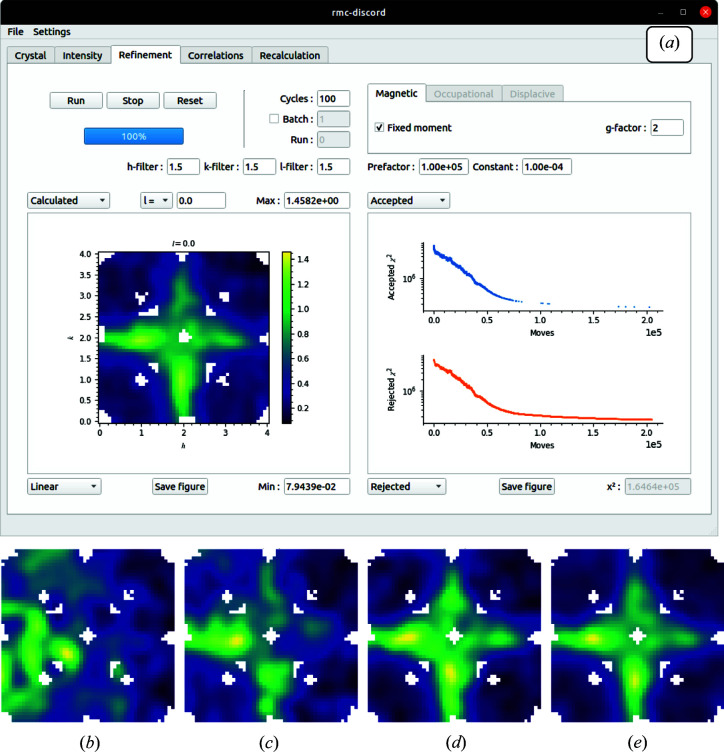
(*a*) The GUI ‘Refinement’ tab showing progress of the calculated intensity during the refinement (*b*)–(*e*) from 0–100%. The moves that are accepted and rejected are displayed in auxiliary monitor plots that update automatically along with the intensity throughout the refinement.

These accepted and rejected moves can be monitored in a plot window that is automatically updated throughout the refinement and gives the goodness-of-fit value (χ^2^) of accepted and rejected moves. This window offers several other diagnostic plots, including overall χ^2^, energy, temperature and scale factor. In addition, the refined intensity plot can be compared with the experimental data set. Other parameters relevant to each refinement type (magnetic, occupational, displacive) can be specified before running the refinement. A batch job can be specified to give multiple sequential refinements that can be used to improve the refinement statistics by averaging. An example of a completed refinement is shown in Fig. 14 ▸, which gives the final screenshot in (*a*) and an illustration of the refinement progress over time in (*b*)–(*e*). The tab allows for user interaction during a refinement and the parameters may be updated as it progresses. At any time, the refinement can be stopped or reset with new parameters as needed. Further capabilities to control the refinement are expected additions, including combined occupational/displacive disorder of polyhedra.

### 4.4. ‘Correlations’ tab

The ‘Correlations’ tab provides the ability to calculate both spherically averaged and three-dimensional correlations for occupational, displacement and magnetic disorder. A cutoff distance is employed to limit the search of pairs using the underlying *k*D tree. The distance tolerance is also employed to account for rounding error in distances. Too tight a tolerance may identify more pairs than actually exist by subtle differences in decimal places introduced by rounding error. Too loose a tolerance may not distinguish real differences in bond lengths. Support for size-effect correlations will also be implemented in future releases.

**Figure 15:**
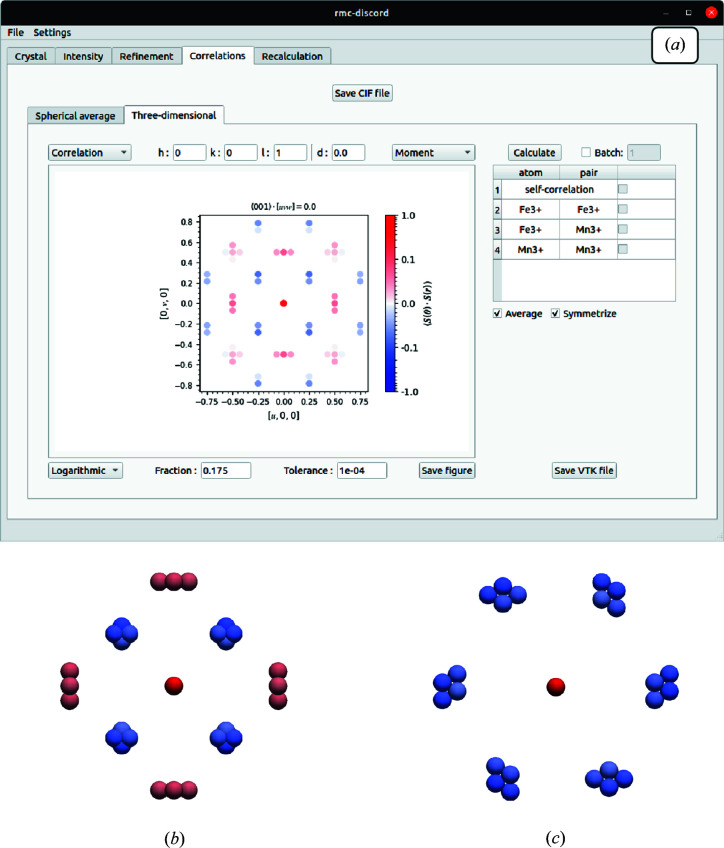
(*a*) The GUI ‘Correlations’ tab showing calculated three-dimensional magnetic spin-pair correlations. The figure also displays visualizations of nearest neighbors in *ParaView* viewed (*b*) along the [001] axis, showing those observed in the *z*/*a* = 0 plane corresponding to the 3D-ΔPDF, and (*c*) along the [111] axis, showing those corresponding to the nearly hexagonal frustrated network.

In the case of spherically averaged correlations, a line plot up to a specified radial cutoff distance provides a simple way to identify the strongest correlations. Three-dimensional correlations are visualized as two-dimensional slices through the specification of lattice planes. Future capabilities will allow one-dimensional plots along particular directions. For any pair correlations, particular atom-pair types may be separated out to help visualize the strength of their interaction. This is especially important if two or more different pairs share the same separation vectors. Without accounting for pair identity, they cannot be distinguished if they have the same separation. However, the correlations may be recalculated with these pairs that are the same distance apart averaged together. In bixbyite, several Fe–Fe, Fe–Mn and Mn–Mn pairs share the same separation vector. Shown in Fig. 15 ▸(*a*) are the calculated three-dimensional correlations at *z*/*a* = 0 with all pairs averaged together. The calculated spherical average and three-dimensional correlations may be exported as a comma-separated values (CSV) file or as visualization toolkit (VTK) files, respectively, for further processing. For example, Figs. 15 ▸(*b*) and 15 ▸(*c*) show the three-dimensional correlations visualized with the external viewer *ParaView* (Ayachit, 2015 ▸) along two different orientations. Further correlation calculations are expected additions, including the occupancy–displacement pair correlations. The capability to map the calculated correlations to the average structure and visualize them is also planned.

### 4.5. ‘Recalculation’ tab

**Figure 16:**
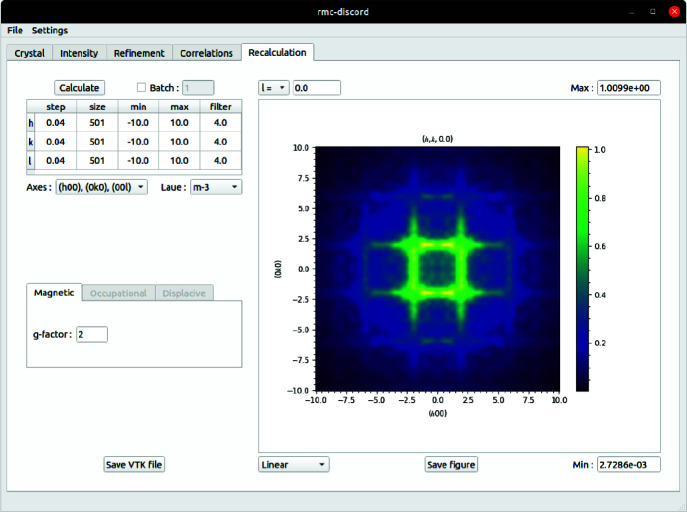
The GUI ‘Recalculation’ tab showing the recalculated scattering intensity. The recalculation is symmetrized using the underlying 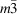 Laue symmetry of bixbyite. The resulting pattern compares favorably with the full-volume experimental data set despite using a smaller region for refinement.

Having obtained the refined structure, it is possible to recalculate the diffuse-scattering pattern back over the original (or any) size with resolution higher than that used for refinement. It is then possible to examine and compare with the original experimental data set. During this recalculation, the symmetry in reciprocal space may optionally be restricted. The Laue symmetry can be selected or inferred from the loaded CIF file in the ‘Crystal’ tab. Of course, the diffuse-scattering pattern is not required to have the same Laue symmetry of the average structure but instead may have symmetry that is lower. For the case of bixbyite as shown in Fig. 16 ▸, the symmetry of the diffuse scattering appears to have the same Laue symmetry as the average structure 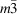 . The recalculated scattering pattern can be exported to a VTK file for external visualization in *ParaView*.

## 5. Program availability

The program is built to accommodate new features based on user needs and evolving data approaches. The source code is located on GitHub for download. An accompanying web site containing tutorials and installation instructions is also available: https://zjmorgan.github.io/rmc-discord/. The content includes example refinements of classical disordered systems with data and instructions to help new users get started. This includes disorder on triangular, honeycomb, kagome and pyrochlore lattices.

## 6. Limitations and guidance

This program is only suited for crystalline materials and not amorphous or powder materials. Programs like *RMCProfile* (Tucker *et al.*, 2007 ▸) can handle those cases. The maximum correlation length that can be resolved in the refinement along any supercell dimension is half the size of the corresponding supercell length. This also determines the sharpness of the features in reciprocal space. A three-dimensional volume data set is required, which appropriately weights measurement scans together and normalizes for Lorentz and spectrum corrections. To refine the diffuse scattering only, Bragg peaks can be removed, but information about the absolute scaling of magnetic moments, site occupancies and atomic displacements is lost in the overall scale factor. Diffuse magnetic scattering data should be isolated by subtracting from the low-temperature data a higher-temperature data set (Paddison *et al.*, 2013 ▸; Roth *et al.*, 2018 ▸). Alternatively, background subtractions should be performed from empty instrument measurements scaled to the same incident flux as the normalization data (Michels-Clark *et al.*, 2016 ▸). Although powder lines that do not overlap with diffuse-scattering features do not affect the overall refinement as demonstrated with the Ba_3_Co_2_O_6_(CO_3_)_0.7_ example, it is generally advised to exclude them in areas where there is severe contamination with the diffuse-scattering features.

## 7. Future development

An opportunity for the single-crystal RMC approach is a co-refinement of the diffuse and Bragg scattering to obtain both the short- and long-range ordering (Dove *et al.*, 2002 ▸; Proffen & Kim, 2009 ▸). By removing Bragg peaks, average structure information is lost that may contain critical information about the origin of the disorder. Future implementations will include refining the integrated Bragg intensities which would give average structure constraints, guiding the RMC refinement and ensuring consistency over the full range of the collected data. It would also allow the absolute values for moments, occupancies and displacements to be obtained. For complete volume data sets, 3D-PDF analysis could also be incorporated prior to performing RMC refinements of the total scattering, like that implemented by the *Yell* program. Previous studies demonstrated this approach using PDF analysis before RMC refinement with powder data (Whitfield *et al.*, 2016 ▸). Also, the data-preprocessing step would be simplified by eliminating the need for Bragg-peak removal. This approach may benefit magnetic structure analysis for magnetically dilute systems and may also allow for analysis of structures with twinning.

Future work is planned to improve upon the molecular-structure-factor work, extending the displacement and rotation of rigid structures to account for networks of corner-linked polyhedra with constraints. This will enable the diffuse-scattering refinement of networks with rigid units. Similarly, the use of *Z* matrices for molecules (Goossens *et al.*, 2011 ▸) is another capability that could be implemented, allowing for the flexibility and rigidity of various portions of a molecule to be specified. An interface to interactively build and visualize polyhedra within the GUI is also planned.

Recently, a formalism for interpreting diffuse scattering based on a disordered superspace approach has been introduced for systems with substitutional disorder (Schmidt & Neder, 2019 ▸). Physical-space structures are realized that generate diffuse-scattering patterns with specified width and location in reciprocal space. Compared with RMC where the interpretation of diffuse scattering is in terms of correlation parameters, this method has the potential to yield a structure solution with more physical meaning at a reduced computational cost, allowing direct interpretation of the diffuse-scattering features in terms of the modulation functions. An innovation for RMC that considers the symmetry of disorder is highly desired. Symmetry analysis is key to understanding distortion modes that are responsible for unusual properties, for example in layered perovskites that undergo switching from positive to negative thermal expansion (Senn *et al.*, 2016 ▸). One opportunity is to utilize *ISODISTORT* to identify distortion symmetries from the input CIF file of the parent structure (Campbell *et al.*, 2006 ▸). This could be used to restrict the possible distortions of individual atoms in disordered crystals that undergo phase transformations, to speed up RMC refinements and improve the analysis of these systems.

## 8. Conclusions

A complete and user-friendly RMC refinement program has been developed to analyze single-crystal diffuse-scattering data. The program addresses each of the three disorder types: occupational, displacive and magnetic. It is optimized for performance and is accompanied by a user-friendly graphical interface that allows a user to load in collected data, process them efficiently, perform a refinement and extract correlations that describe the disorder within the crystal. It effectively demonstrates the extraction of correlations from single-crystal diffuse-scattering data in two different scientific user cases. In mineral bixbyite, RMC offers richer detail of the spin-pair antiferromagnetic correlations than the magnetic 3D-ΔPDF by distinguishing the subtle differences in separation vectors between first- and second-nearest neighbors of Fe–Mn and Mn–Mn pairs. In the triangular lattice system Ba_3_Co_2_O_6_(CO_3_)_0.7_, RMC reveals no clustering of vacant CO_3_ sites along *c*-axis chains, and the occupied CO_3_ sites move toward their closest neighboring vacant sites along the chain to relax strain induced by electrostatic interactions.
